# Combining *Metarhizium anisopliae* with spinetoram synergistically suppresses *Frankliniella occidentalis* via reciprocal enhancement

**DOI:** 10.1080/21505594.2026.2753272

**Published:** 2026-10-29

**Authors:** Lu Yu, Jing Chen, Yuxian Xia, Biao Guo, Yi Yu, Guoxiong Peng

**Affiliations:** a School of Life Sciences, Chongqing University, Chongqing, China; b Chongqing Engineering Research Center for Fungal Insecticide, Chongqing, China; c Chongqing Shapingba District Plant Protection and Inspection Station, Chongqing, China; d Shandong Key Laboratory for Green Prevention and Control of Agricultural Pests, Institute of Plant Protection, Shandong Academy of Agricultural Sciences, Jinan, China

**Keywords:** *Frankliniella occidentalis*, *Metarhizium anisopliae*, synergism, chemical pesticide, reciprocal enhancement

## Abstract

The combination of insecticides and entomopathogenic fungi, valued for both high efficacy and environmental compatibility, has become an important strategy for sustainable pest management, and their efficient combinations and underlying mechanisms have emerged as key research topics. *Metarhizium anisopliae* CQMa421 combined with compatible spinetoram exerts synergistic insecticidal activity against *Frankliniella occidentalis*, enhancing pest susceptibility to spinetoram, suppressing second-generation nymph, pupa and adult populations, and reducing the overall pupation rate. Mechanistically, the combined treatment upregulated the resistance target *nAChRα6* and disrupted the activity of resistance-associated detoxifying enzymes (GST, CarE, POD, and CYP450) compared with spinetoram, while downregulating immune genes (*GNBP1/2*, *Spätzle1/2*, *MyD881*, *Defensin*, and *PPO*) compared with CQMa421. Accordingly, the median times for spore germination (GT_50_) and appressorium formation (AT_50_) of CQMa421 on the thrips cuticle were significantly shortened. Field trials showed that the combination (1 × 10^7^ conidia/mL CQMa421 + 10 mg/L spinetoram) provided superior rapid and persistent control (90.67% at 3 d; 86.33% at 14 d) compared with spinetoram at 20 mg/L (74.33% and 43.33%, respectively). Consistently, in the continuous control trial (six applications of the combined treatment vs nine of spinetoram), the combined treatment maintained significantly lower thrips population densities during peak infestation. This study shows that CQMa421 combined with spinetoram disrupts detoxification enzyme activity, enhances pesticide sensitivity in *F. occidentalis*, and facilitates early cuticular colonization by CQMa421, achieving synergistic control efficacy through reciprocal enhancement. This synergism was validated under field conditions. These findings provide a theoretical and practical basis for effective, safe field fungal-chemical control.

## Graphical abstract

**Figure:**
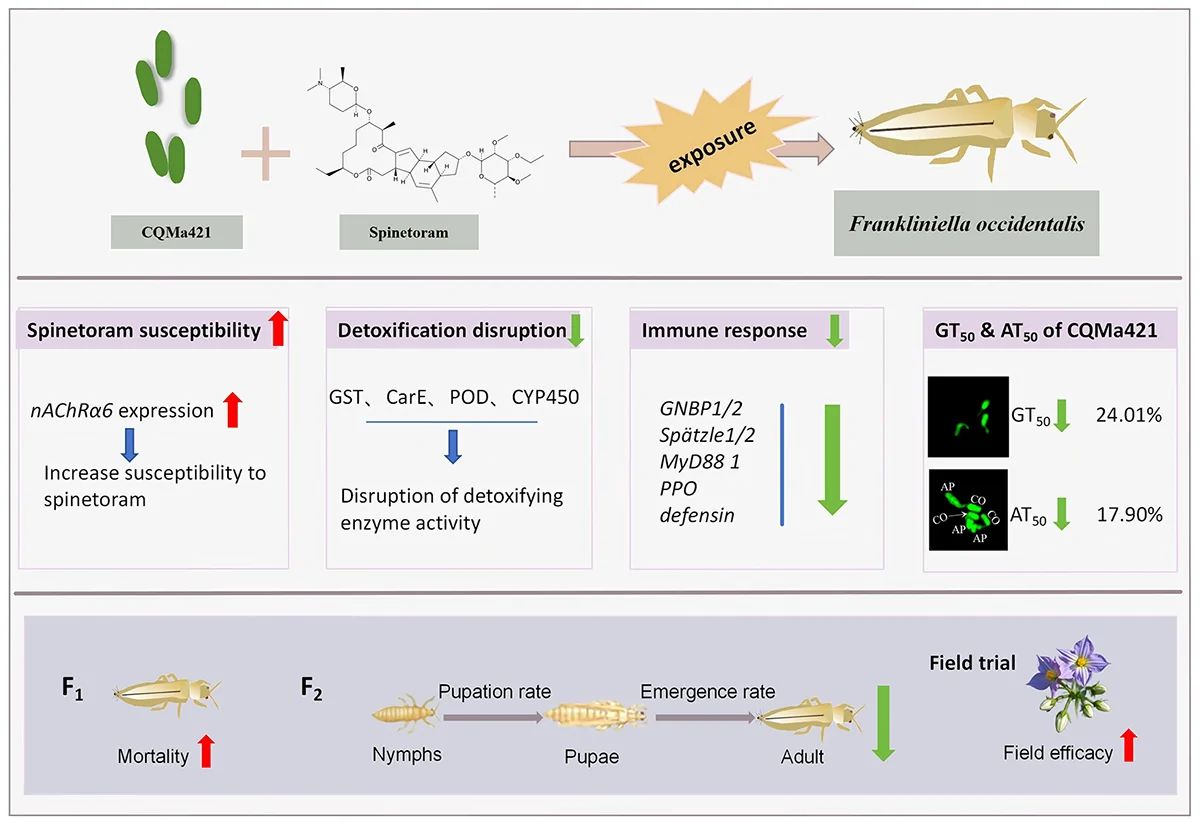

## Introduction

Western flower thrips (*Frankliniella occidentalis*, Pergande) (Thysanoptera: Thripidae) is a polyphagous pest native to North America, causing substantial economic losses in crops such as rice, cotton, and eggplant [1,2]. Its small size, rapid development, high fecundity, and cryptic habits make effective control difficult. This species damages plants through feeding and oviposition within tissues and transmits numerous economically important plant viruses [3,4]. Currently, chemical insecticides remain the primary tool for controlling *F. occidentalis* [5]. However, the intensive use of broad-spectrum insecticides has led to resistance in this pest to key active ingredients, including spinetoram (1487.7-fold), emamectin benzoate (356-fold), and imidacloprid (127.8-fold) [6–8]. To date, *F. occidentalis* has developed resistance to 32 insecticide active ingredients (www.pesticideresistance.org, 2026). These challenges, coupled with growing concerns over the non-target effects and food safety risks of intensive pesticide use, underscore the need for more sustainable, effective solutions for *F. occidentalis* control.

Entomopathogenic fungi represent a promising option due to their broad host range, epizootic potential, and persistence in soil [9]. Infection is initiated when fungal appressoria generate mechanical pressure and secrete enzymes to penetrate the host cuticle, followed by proliferation within the hemocoel and secretion of insecticidal toxins that ultimately kill the host [10,11]. Several entomopathogenic fungi show potential for controlling *F. occidentalis*. *Lecanicillium attenuatum* and *L. cauligalbarum* infect *F. occidentalis*, exerting a sublethal effect by disrupting microbial homeostasis and thereby providing sustained suppression of pest populations [12]. Under controlled greenhouse conditions, *Beauveria bassiana* effectively suppresses *F. occidentalis* populations [13], while *Metarhizium anisopliae* V275 achieves 70–90% control of pupal stages [14]. However, the field performance of entomopathogenic fungi is highly dependent on environmental conditions, especially temperature and humidity, and their relatively slow mode of action often permits infected hosts to sustain feeding and cause crop damage before succumbing to infection [15,16]. Accordingly, strategies to accelerate fungal pathogenesis or sensitize hosts to fungal infection are highly sought after.

The combined application of entomopathogenic fungi and chemical insecticides offers a promising avenue to address these challenges. This synergism typically stems from complementary modes of action and reciprocal potentiation of biological effects in the target host [17]. Chemical insecticides act rapidly by targeting specific physiological and neural sites in insects [18], while entomopathogenic fungi exert toxicity via a sequential process of cuticle penetration, in vivo colonization, toxin production, and disruption of host homeostasis [19]. The two agents differ significantly in their modes of action, providing a potential basis for synergistic effects. However, the physiological and molecular mechanisms underlying this synergy remain to be fully elucidated and warrant further in-depth investigation. Our laboratory originally isolated and characterized the broad-spectrum entomopathogenic fungus *M. anisopliae* CQMa421, which has been commercially registered for controlling over 10 crop pests (PD20171744, http://www.icama.org.cn/zwb/dataCenter?hash=reg-info). Integrating CQMa421 with the chemical insecticide thiamethoxam significantly enhanced control efficacy against the brown planthopper (*Nilaparvata lugens*) in rice [20]. In combination with reduced rates of 50% flonicamid, 10% flonicamid amide, or 50 g/L diflubenzuron, CQMa421 exhibited significant synergistic effects against aphids on cotton seedlings [21]. More recently, CQMa421 combined with emamectin benzoate significantly increased mortality and suppressed reproduction in fall armyworm (*Spodoptera frugiperda*) [22]. These results provide a foundation for future research on the combined application of CQMa421 and chemical insecticides and their underlying synergistic mechanisms.

Preliminary studies indicate that *M. anisopliae* CQMa421 shows high efficacy against *F. occidentalis* populations [23]. To identify compatible chemical partners for CQMa421, we first screened its biological compatibility with multiple conventional insecticides and identified spinetoram as a promising candidate due to its high compatibility. As a primary thrips-control insecticide, spinetoram faces widespread high-level resistance in *F. occidentalis* populations, constraining its long-term efficacy as a standalone treatment. The favorable compatibility and distinct modes of action make the combined treatment an ideal model to investigate complementary and synergistic interactions between chemical insecticides and entomopathogenic fungi. In this study, we systematically assessed the synergistic activity of this combination against *F. occidentalis*, explored the involvement of synergistic disruption of host defense, detoxification, and metabolic pathways in this potentiation, and validated the efficacy under field conditions. Through compatibility screening, laboratory virulence assays, mechanistic dissection, and field verification, this study provides a theoretical basis for the combined application of entomopathogenic fungi and chemical pesticides.

## Material and methods

### Test strain and target pest

The fungal strain *M. anisopliae* CQMa421 (China General Microbiological Culture Collection Center, accession No. CGMCC 4608) used in this study was provided by the Genetic Engineering Research Center, Chongqing University. The strain was reactivated on 1/4 SDAY medium (0.25% peptone, 0.5% yeast extract, 1.0% glucose, 1.8% agar) and incubated in darkness at 27 ± 1°C for 15 d. Mature conidia were collected using a sterile spatula and reserved for subsequent experiments.

The experimental population of *F. occidentalis* was sourced from the Institute of Entomology, Shandong Academy of Agricultural Sciences. Thrips were maintained at 27 ± 1°C, 75% ± 5% relative humidity, and a 14:10 h (L:D) photoperiod. Fresh pods of *Phaseolus vulgaris* L. served as food and were replaced every 2–3 d, with the excised beans placed in clean rearing containers for subsequent egg hatching and nymph rearing. *P. vulgaris* L. was collected from the experimental farm of Chongqing University in Shapingba District, Chongqing.

### Compatibility test of CQMa421 with chemical pesticides

#### Determination of the effects of 12 commonly used insecticides on the germination of CQMa421 spores

*M. anisopliae* CQMa421 conidial suspension (2 × 10^7^ conidia/mL) and chemical insecticide solution (2× recommended field concentration) were prepared in 0.1% Tween-80. The two solutions were mixed at a 1:1 (v/v) ratio and allowed to stand at 27 ± 1°C for 5 h. After vortexing, 100 μL of the mixture was spread evenly on a 1/4 SDAY plate and incubated at 27 ± 1°C for 24 h. After incubation, agar blocks (0.5–1 cm^2^) were randomly excised and microscopically examined at 400× for spore germination. Spore germination was defined as a germ tube length exceeding the spore radius or half its longest dimension. For each treatment, three plates were randomly selected, and 200 conidia were counted per plate. A conidial suspension without pesticide was prepared as the blank control.

### Determination of the effect of spinetoram on the growth of CQMa421 colonies

A spinetoram stock solution (200 mg/L) was prepared in sterile distilled water and filter-sterilized through a 0.22 μm membrane. Molten 1/4 SDAY (900 mL) was cooled to 50–55°C and combined with 100 mL sterile spinetoram stock solution to 20 mg/L. The medium was thoroughly mixed and dispensed into 9 cm diameter Petri dishes. The culture medium was allowed to cool and solidify until the surface was dry before use. A 1/4 SDAY plate without spinetoram was used as the control. For each treatment, three plates were randomly selected. A 2 µL aliquot of the conidial suspension (1 × 10^7^ conidia/mL of *M. anisopliae* CQMa421) was inoculated at the center of each plate. After air-drying, plates were incubated inverted at 27 ± 1°C. Colony growth was monitored every 24 h by measuring diameter and capturing records.

### Bioactive synergistic enhancement research

Female adult *F. occidentalis* (3–5 d) were used in bioassays with a modified apparatus [24]. The apparatus has three stacked 11 × 9 cm acrylic plates with a central 4-cm-diameter circular exposure chamber. Ventilation holes (2 mm diameter) were drilled into the side walls, each covered with a copper mesh screen (0.212 mm mesh size) to facilitate adequate airflow while preventing insect escape (Figure 1).

**Figure 1.**
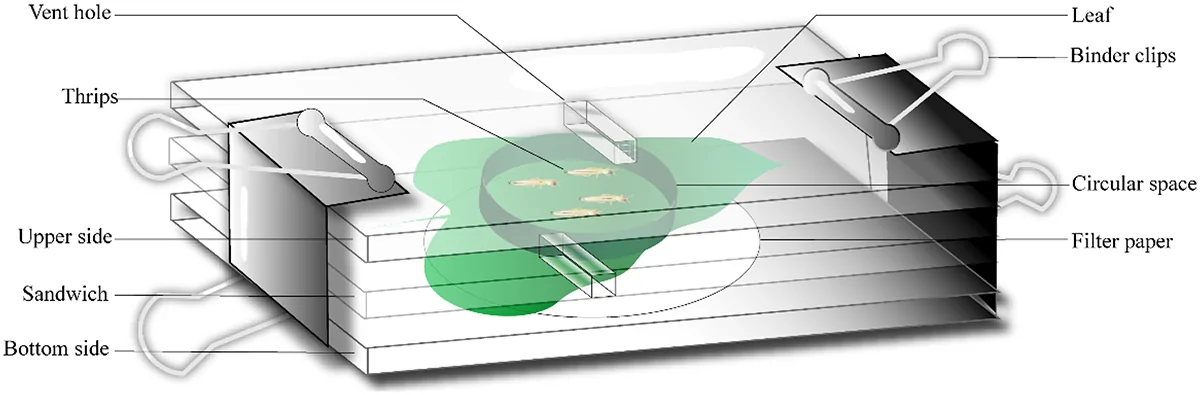
Schematic diagram of experimental apparatus for *F. occidentalis* bioassays.

Based on preliminary assays and field application considerations, CQMa421 was tested at 1 × 10^6^ and 1 × 10^7^ conidia/mL. The concentration of 1 × 10^7^ conidia/mL corresponds to the recommended field dose, whereas 1 × 10^6^ conidia/mL served as a low-dose treatment to characterize the dose-dependence of synergistic effects. According to Table 2, spore suspensions of *M. anisopliae* CQMa421 (1 × 10^6^ and 1 × 10^7^ conidia/mL), spinetoram solutions (0.01875, 0.0375, 0.075, 0.15, and 0.3 mg/L), and their combinations were prepared. A 0.1% Tween-80 solution was used as the blank control. Filter paper was placed in the chamber and 1 mL distilled water added to maintain leaf moisture. A *P. vulgaris* L. leaf disc was placed on the moistened filter paper, and the three-layer chamber was assembled. Using a Burkard spray tower (Burkard Manufacturing, UK), 200 μL of each test solution was uniformly applied to the exposure arena. Thirty healthy adult females were selected and transferred to 1.5 mL centrifuge tubes. Following CO_2_ immobilization, the thrips were introduced into the exposure chamber. The apparatus was maintained in a controlled-environment incubator, and thrips mortality was assessed daily. The experiment was replicated three times.

### *Effects of combined treatment on reproduction and development of* F. occidentalis

Three treatment groups were included: CQMa421 conidial suspension (1 × 10^7^ conidia/mL), a sublethal concentration of spinetoram (0.03 mg/L), and their combination (1 × 10^7^ conidia/mL +0.03 mg/L). A 0.1% Tween-80 solution served as the blank control. Newly emerged adults were maintained separately by sex for 3–5 d prior to use. From each treatment group, 30 healthy adults were selected and treated with 50 μL of the corresponding test solution following the procedure described in the bioassay method above. One hour post-treatment, 50 females and 50 males were placed in glass bottles with fresh *P. vulgaris* L. pods for mating and oviposition. Fresh *P. vulgaris* L. pods were replaced daily, and excised pods were transferred to clean rearing jars for nymph hatching and development. *F. occidentalis* pupae are non-feeding and largely immobile, with prominent wing buds and antennae directed anteriorly or curved dorsally over the head. Adults have two pairs of fully developed fringed wings and exhibit active crawling and flight behavior. Following hatching, second-generation nymphs, pupae, and adults were counted daily to assess reproductive output and developmental performance. Pupation rate was calculated as the percentage of nymphs that successfully developed into pupae, and adult emergence rate was calculated as the percentage of pupae that successfully eclosed into adults. The experiment was repeated three times.

### Enzyme activity assay

Previous studies have confirmed that carboxylesterase (CarE), glutathione S-transferase (GST), peroxidase (POD), and cytochrome P450 (CYP450) are key mediators of xenobiotic detoxification, antioxidant defense, and insecticide resistance in insects [25]. To elucidate the impact of the combined treatment on the detoxification enzyme system of *F. occidentalis*, we assayed the activities of these four enzymes in the present study. Three treatment groups were established as described above, with 0.1% Tween-80 serving as the blank control. Adult female western flower thrips were treated in accordance with the bioassay method, and surviving insects were collected at 24, 36, 48, 60, and 72 h post-treatment. Following CO_2_ anesthesia, the test insects were transferred to 1.5 mL centrifuge tubes. For each treatment, 0.01 g of surviving *F. occidentalis* was weighed out, flash-frozen in liquid nitrogen, and stored at −80°C, with four biological replicates per treatment. CarE, GST, POD, and CYP450 activities were assayed with commercial kits (Suzhou Keming Biotechnology Co., China) according to the manufacturers protocols. CarE, GST, and POD activities measurements were performed on a UV–Vis spectrophotometer (Model V1200, China) at three wavelengths (450, 340, and 470 nm). CYP450 activity was quantified fluorometrically with excitation at 380 nm and emission at 450 nm.

### Gene expression assay

To elucidate the molecular mechanisms by which combined treatment modulates spinetoram susceptibility and host immunity in *F. occidentalis*, we selected *nAChRα6*, which encodes the target subunit of spinetoram [26]. Immune-related genes of the Toll pathway, including *GNBP1/2*, *PPO*, *Defensin*, *Spätzle1/2*, and *MyD88 1/2*, were used to evaluate host-immune responses to fungal and combined treatments, enabling simultaneous detection of insecticide-triggered responses and fungus-induced immune reactions. Three treatment groups were established as described above, with 0.1% Tween-80 used as the blank control. Adult female western flower thrips were treated in accordance with the bioassay method, and surviving insects were collected 48 h post-treatment. For each biological replicate (*n* = 3), 50 adult females were pooled and processed. Samples were immediately transferred to pre-chilled tubes with steel beads, snap-frozen in liquid nitrogen, and stored at −80°C. The tissue was ground to a fine powder using a tissue grinder. Total RNA was extracted with the BIOER RNA Extraction Kit (BG0050-L) according to the manufacturer’s instructions. RNA quality was assessed via 2% agarose gel electrophoresis. First-strand cDNA was synthesized from 1 μg total RNA using PrimeScript™ RT Master Mix (Takara, RR047A); qPCR was performed with 2× SYBR Green qPCR Mix (BIOER, BG0014). The relative expression levels of each gene were calculated using the 2^−ΔΔ^CT method. All primer pairs exhibited amplification efficiencies between 90% and 110% with R^2^ values > 0.98, satisfying the standard requirements for quantitative analysis using the 2^−ΔΔ^CT method. Primers for each gene and the housekeeping gene *β-actin* (Table S1) were synthesized by Shanghai Sangon Biotech.

### *Assay for spore germination and appressorium development on the surface of* F. occidentalis

CQMa421 expressing GFP (obtained via Agrobacterium transformation with a GFP-carrying recovery vector) was mixed with spinetoram. The combined treatment comprised 1 × 10^7^ conidia/mL and 0.03 mg/L, with CQMa421 (1 × 10^7^ conidia/mL) as control. Adult female *F. occidentalis* were inoculated as described in the bioassay method above. Samples were collected every 4 h, starting at 6 h post-inoculation. Live adults were briefly immobilized with CO_2_ and placed onto pre-chilled microscope slides. Slides were mounted with ddH_2_O, and CQMa421 germination and appressorium formation on the insect surface were examined under a fluorescence microscope. Approximately 100 spores per sample were observed, and the procedure was repeated three times.

### Field trial

Field trials were conducted in a level, uniformly infested eggplant field in Bishan, Chongqing, China. The trial comprised five treatments: (1) the combination of CQMa421 (1 × 10^7^ conidia/mL) and spinetoram (10 mg/L); (2) spinetoram at 10 mg/L; (3) spinetoram at 20 mg/L; (4) CQMa421 at 1 × 10^7^ conidia/mL; and (5) a 0.05% Tween-80 control. Fifteen plots (each 10 m × 10 m) were established, separated by 1 m‑wide buffer strips. For each treatment, three plots were randomly selected as replicates. The treatments were applied using an electric sprayer (China Lano Co., Ltd.) at a rate of 750 L/ha. *F. occidentalis* populations were assessed at 1, 3, and 14 d pre- and post-treatment per Li et al [23].

Field demonstration trials were conducted in eggplants with relatively uniform *F. occidentalis* infestation. The trial had two treatments: spinetoram (20.0 mg/L) and its combination with CQMa421 (1 × 10^7^ conidia/mL +10.0 mg/L spinetoram). The trial consisted of 6 plots (approximately 1,000 m^2^ each), with three replicates per treatment. *F. occidentalis* density in each treatment area was assessed per Li et al [23].

### Statistical analysis

To assess synergy between *M. anisopliae* CQMa421 and spinetoram, combined treatment data were analyzed with the Independent Action model. The model computes theoretical additive mortality for the mixture based on empirically derived mortality rates of single-agent treatments. A chi-square (χ^2^) test was applied to classify effects as additive, synergistic, or antagonistic, an approach well suited to our experimental design and data properties. Per Nishimatsu and Jackson (1998) [27], the interaction was assessed by the chi-square test of expected and observed mortality and classified as additive, synergistic, or antagonistic.

Under the hypothesis of additivity (i.e., no interaction between agents), the expected mortality rate (M_e_) was calculated as follows:

Me=Ma+Mb×(1−Ma)

$$\begin{array}{rl} & \chi =[(\mathrm{Mab}-\mathrm{Me})\times 100]\times [(\mathrm{Mab}-\mathrm{Me})\times 100]\\ & /(\mathrm{Me}\times 100) \end{array}$$

Me is expected additive mortality; Ma, Mb, and Mab are observed mortalities for CQMa421, spinetoram, and their combination, respectively. The *p*-value was determined by referring to the chi-squared distribution table with 1 degree of freedom. An additive effect was concluded when χ^2^ < 3.84. An antagonistic effect was indicated when χ^2^ > 3.84 and Mab < Me.

All mortality data were first adjusted for comparison (Abbott, 1987), and percentages were converted to ratios (i.e., 0–100% converted to 0–1):

$$\begin{array}{rl} & \mathrm{Mortality}\;\mathrm{adjusted}(\%)=[(\mathrm{Mortality}\;\mathrm{treatment}-\\ & \mathrm{Motality}\;\mathrm{control})/(100-\mathrm{Mortality}\;\mathrm{control})]\\ & \times 100\% \end{array}$$

LC_50_ values and their corresponding 95% confidence intervals (95% CI) of spinetoram for each treatment were calculated using GraphPad Prism software. The LC_50_ reduction ratio was calculated as follows:

LC_50_ reduction ratio = LC_50_ of spinetoram/LC_50_ of spinetoram in the combined treatment.

Relative efficacy (%) in field trials was calculated based on *F. occidentalis* population density on eggplant using the following formula (4) [28]:

[1−(DtjDc0/Dt0Dcj)]×100%

Dc0 and Dcj are thrips populations in control on the pre-treatment day and on day j post-application; Dt0 and Dtj are the corresponding populations in plots treated with CQMa421 or spinetoram.

All statistical values are expressed as mean ± standard deviation (SD). For mortality and other percentage-based data, percentage values were first converted to proportions (0–1) and then arcsine square-root transformed prior to statistical analysis. The normality and homogeneity of variance of the transformed data were assessed before parametric analyses. Student’s t-test was used for comparisons between two treatment groups, whereas one-way analysis of variance (ANOVA) followed by Tukey’s post hoc test was used for comparisons among three or more groups. Synergistic effects of the insecticide–fungus combination were evaluated using the Independent Action (IA) model by comparing the observed mortality with the theoretically predicted mortality. Significance levels were indicated as follows: *p < 0.05, **p < 0.01, ***p < 0.001, ****p < 0.0001; ns denotes no significant difference. Statistical analyses were performed using GraphPad Prism 9.0 (GraphPad Software, San Diego, CA, USA).

## Results

### Screening chemical pesticides compatible with CQMa421

Among the 12 chemical pesticides tested against thrips, we found that 11 significantly inhibited the spore germination rate of CQMa421 (F_12,26_ = 26.65, *p* < 0.001, *n* = 3). In the 6% spinetoram treatment group, the germination rate of CQMa421 spores reached 97.50% ± 0.50% at 16 h, showing no significant difference compared to the control group (97.67% ± 0.58%), demonstrating that it exerts no inhibitory effect on the germination of CQMa421 spores (Table 1). Colony comparison revealed that CQMa421 exhibited faster growth on plates supplemented with 6% spinetoram (Figure 2(A)), with significantly larger colony diameters observed after 2–4 d of incubation compared to the control group (Figure 2(B)). These results indicate that spinetoram promotes the growth of CQMa421.

**Figure 2.**
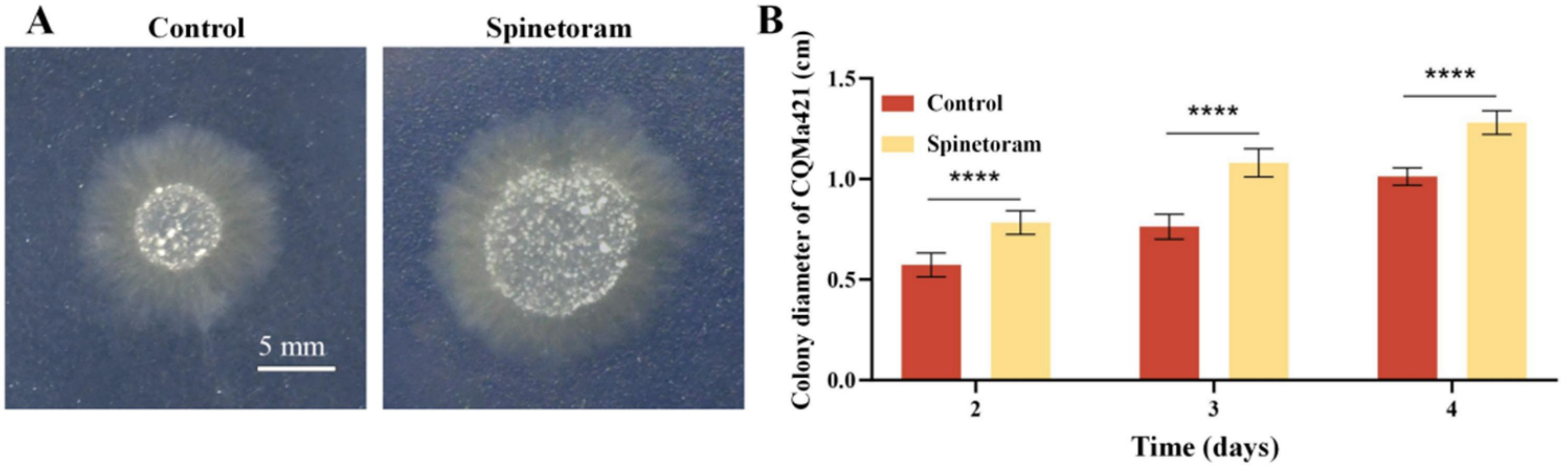
CQMa421 colony growth analysis.

(A) Morphology of CQMa421 colonies. (B) Diameter of CQMa421 colonies. *Indicates statistically significant difference, *t-test*: ****p < 0.0001.

**Table 1.** Effect of pesticides on spore germination rate of CQMa421.

	Ingredients	Recommended concentration (mg/L)	Manufacturers	Germination Rate (%)
1	6% Spinetoram SC	20.00 mg/L	Dow Agrosciences	97.50 ± 0.50a
2	25% Thiamethoxam WG	104.17 mg/L	Rui Defeng, China	90.67 ± 2.31b
3	21% Thiamethoxam SC	70.00 mg/L	Syngenta	91.50 ± 1.80b
4	10% Cyantraniliprole SC	66.67 mg/L	FMC	86.67 ± 1.53c
5	5% Emamectin benzoate ME	3.75 mg/L	Zhong Xun, China	92.00 ± 2.65b
6	5% Emamectin benzoate ME	3.75 mg/L	Jing Bo, China	83.67 ± 2.08d
7	5% Emamectin benzoate ME	3.75 mg/L	Jun Xing, China	74.00 ± 1.73e
8	10% Broflanilide SC	26.67 mg/L	BASF	91.33 ± 2.08b
9	20% Acetamiprid SC	53.33 mg/L	Wei Yuan, China	86.33 ± 1.15c
10	20% Dinotefuran SC	133.33 mg/L	Mitsui Group	89.00 ± 2.65b
11	25% Deltamethrin EC	166.67 mg/L	Hui Feng, China	87.00 ± 3.61c
12	10% Spinosad SC	41.67 mg/L	Hua Xing, China	92.33 ± 2.08b
13	Water			97.67 ± 0.58a

Note: SC = Suspension concentrate; WG = Water-dispersible granules; ME = Microemulsion; EC = Emulsifiable concentrate. Different letters indicate significant differences between treatments (*p* < 0.05).

### *Synergistic enhancement effect of CQMa421 and spinetoram on* F. occidentalis

Analysis of Table 2 reveals that CQMa421 at concentrations of 1 × 10^6^ conidia/mL and 1 × 10^7^ conidia/mL generally exhibited synergistic effects when combined with the spinetoram treatment group. Among these, we observed a higher synergistic effect in the CQMa421 combined treatment group at 1 × 10^7^ conidia/mL. Following a three-day combined treatment with 1 × 10^7^ conidia/mL CQMa421 and 0.3 mg/L spinetoram, the mortality rate reached 97% (χ^2^ = 8.48), significantly exceeding the 21% observed in the CQMa421 group and the 65% recorded in the spinetoram group. These results indicate that CQMa421 and spinetoram can interact synergistically to increase mortality of adult *F. occidentalis* under laboratory conditions.

**Table 2.** Synergistic effects on the mean corrected cumulative mortality of adult *F. occidentalis* under laboratory conditions.

Concentration of CQMa421 (conidia/mL)	Time(days)	Concentration of Spinetoram (mg/L)	Mortality				χ^2^	Eggffect
			Ma	Mb	Me	Mab		
		0.01875	0.09	0.07	0.15	0.22	2.69	additive
		0.0375	0.09	0.11	0.19	0.30	5.93	synergistic
	1	0.075	0.09	0.14	0.22	0.36	9.87	synergistic
		0.15	0.09	0.19	0.26	0.41	7.63	synergistic
		0.3	0.09	0.31	0.37	0.49	3.73	additive
		0.01875	0.15	0.15	0.27	0.40	5.87	synergistic
		0.0375	0.15	0.18	0.30	0.46	9.21	synergistic
	2	0.075	0.15	0.22	0.33	0.50	8.53	synergistic
10^7^		0.15	0.15	0.29	0.39	0.58	8.37	synergistic
		0.3	0.15	0.48	0.55	0.74	6.17	synergistic
		0.01875	0.21	0.22	0.38	0.40	0.08	additive
		0.0375	0.21	0.30	0.44	0.47	0.16	additive
	3	0.075	0.21	0.32	0.46	0.65	7.84	synergistic
		0.15	0.21	0.45	0.56	0.76	6.91	synergistic
		0.3	0.21	0.65	0.73	0.97	8.48	synergistic
		0.01875	0.04	0.06	0.10	0.10	0.00	additive
		0.0375	0.04	0.12	0.16	0.15	0.00	additive
	1	0.075	0.04	0.17	0.21	0.28	2.38	additive
		0.15	0.04	0.21	0.24	0.33	2.93	additive
		0.3	0.04	0.28	0.31	0.41	2.99	additive
		0.01875	0.11	0.13	0.22	0.24	0.11	additive
		0.0375	0.11	0.12	0.21	0.29	3.06	additive
	2	0.075	0.11	0.20	0.29	0.37	2.55	additive
10^6^		0.15	0.11	0.27	0.35	0.47	4.78	synergistic
		0.3	0.11	0.44	0.49	0.66	5.25	synergistic
		0.01875	0.14	0.19	0.30	0.34	0.62	additive
		0.0375	0.14	0.22	0.32	0.41	2.52	additive
	3	0.075	0.14	0.32	0.41	0.51	2.38	additive
		0.15	0.14	0.46	0.54	0.69	4.30	synergistic
		0.3	0.14	0.55	0.61	0.79	5.11	synergistic

At 3 d post-treatment, compared with spinetoram alone, the LC_50_ of spinetoram against *F. occidentalis* decreased from 0.1626 mg/L (95% CI: 0.0984–0.4203 mg/L) to 0.0358 mg/L (95% CI: 0.0157–0.0585 mg/L) when combined with *M. anisopliae* CQMa421 at 1 × 10^7^ conidia/mL, representing a 4.54-fold reduction in spinetoram LC_50_. When CQMa421 was applied at 1 × 10^6^ conidia/mL, the LC_50_ of spinetoram in the combined treatment was 0.0566 mg/L (95% CI: 0.0401–0.0766 mg/L). Relative to the corresponding single-agent treatment (0.2152 mg/L, 95% CI: 0.1661–0.3085 mg/L), the LC_50_ showed a 3.80-fold reduction (Table 3). These results demonstrate that CQMa421 significantly enhances the susceptibility of *F. occidentalis* to spinetoram and substantially lowers the spinetoram dosage required to reach equivalent mortality.

**Table 3.** Effects of *M. anisopliae* CQMa421 on the LC_50_ of spinetoram against *F. occidentalis.*

Time (days)	treatment	LC_50_ (mg/L, 95%CI) of Spinetoram	LC_50_ reduction ratio
3	Spinetoram	0.1626 (0.0984–0.4203)	4.54
	Spinetoram + CQMa421 (1 × 10^7^conidia/mL)	0.0358 (0.0157–0.0585)	
3	Spinetoram	0.2152 (0.1661–0.3085)	3.80
	Spinetoram + CQMa421 (1 × 10^6^conidia/mL)	0.0566 (0.0401–0.0766)	

Me denotes the expected mortality rate under additive effects; Ma, Mb, and Mab represent the actual observed mortality rates following treatment with CQMa421 spore suspension, spinetoram, and their combined application, respectively. Should the chi-square value exceed 3.84, this indicates statistically significant differences, demonstrating a synergistic effect in the combined treatment group.

### *CQMa421 exhibits synergistic reproductive suppression of* F. occidentalis *when combined with spinetoram*

Our analysis revealed that, compared with the untreated control, the cumulative numbers of second-generation nymphs, pupae, and adults produced per female were significantly reduced in the CQMa421, spinetoram, and combined treatment groups. These results indicate that these treatments effectively suppressed both oviposition and second-generation population size in *F. occidentalis* (Figure 3(A,C)). We further observed that, compared with the spinetoram group and the CQMa421 group, the combined treatment reduced the cumulative number of second-generation nymphs produced per female by 41.13% and 35.87%, respectively (Figure 3(A)); the number of pupae decreased by 48.02% and 37.50%, respectively (Figure 3(B)); and the number of adults decreased by 55.33% and 64.42%, respectively (Figure 3). These findings demonstrate the enhanced efficacy of the combined treatment in suppressing population development across all life stages.

**Figure 3.**
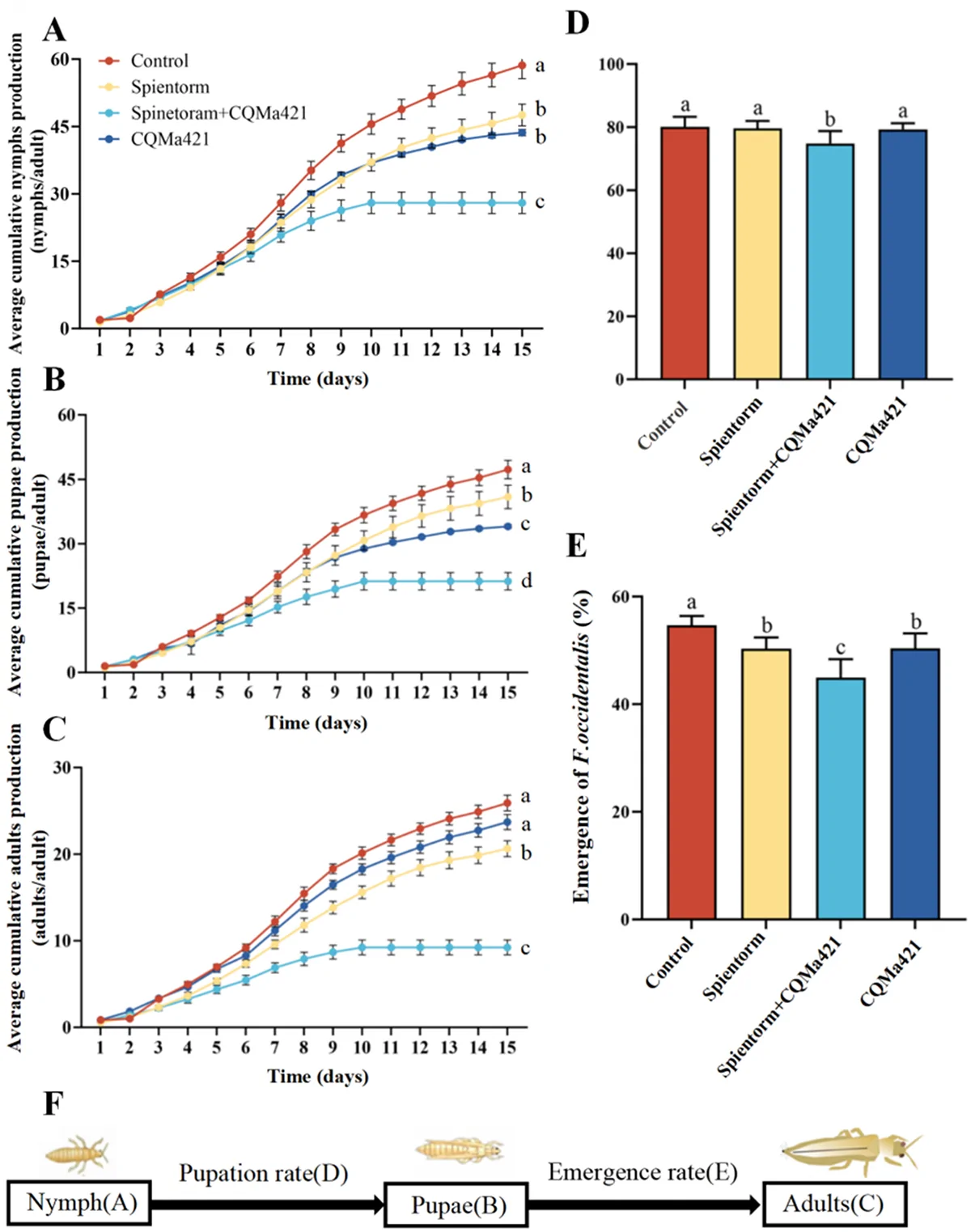
Effects of CQMa421 and spinetoram on the reproductive capacity and development of *F. occidentalis*. (A) Average cumulative number of second-generation nymphs produced per female in each treatment group. (B) Average cumulative number of pupae. (C) Average cumulative number of adults. (D) Pupation rate. (E) Emergence rate. (F) Schematic diagram of *F. occidentalis* in each result. Error bars denote the standard deviation (SD) of the mean. Percentage data were subjected to arcsine square-root transformation before statistical analysis. Different lowercase letters indicate significant differences between groups (Tukey’s honestly significant difference (HSD) test, *p* < 0.05).

We observed that CQMa421 and spinetoram each suppressed the emergence rate of second-generation *F. occidentalis* (Figure 3(E)), but neither treatment affected their pupation rate. Further analysis revealed that the combined treatment significantly suppressed both pupation rate and emergence rate. Compared with the spinetoram group, pupation rate was reduced by 7.37% and emergence rate by 10.02% in the combined treatment; compared with the CQMa421 group, these rates were reduced by 7.06% and 10.10%, respectively (Figure 3). The indicators used to assess the combined effects of the various treatment groups on the reproduction of *F. occidentalis* are shown in Figure 3(F).

The expected and actual numbers of nymphs, pupae, and adults, as well as pupation and emergence rates, were compared using a chi-square test. The results demonstrated that the combined treatment synergistically suppressed second-generation nymph, pupa, and adult populations, in addition to pupation rates. Furthermore, an additive effect on emergence rate suppression was observed for the combined treatment (Table 4).

**Table 4.** Synergistic inhibitory effect of combined treatment with CQMa421 and spinetoram on reproduction and development of *F. occidentalis.*

	Inhibition rate (%)				χ^2^	Effect
	Ma	Mb	Mab	Me		
Nymphs population	25.58	18.93	52.27	39.67	4.01	synergistic
Pupation rate	2.68	0.6	7.93	3.27	6.65	synergistic
Pupae population	28.04	13.48	52.42	37.74	5.73	synergistic
Emergence rate	7.88	9.96	18.22	15.21	0.52	additive
Adult population	8.45	20.33	64.39	27.07	51.46	synergistic

Me denotes the expected value of the summation and inhibition rate; Ma, Mb, and Mab respectively denote the inhibition rates observed when treated with CQMa421 spore suspension, spinetoram, and the combination of both.

### *Effects of spinetoram and CQMa421 on neurological target genes, immune responses, and metabolic enzymes in* F. occidentalis

Mutations in the α6-nicotinic acetylcholine receptor (*nAChRα6*) gene are known to reduce its binding capacity with spinetoram, thereby conferring resistance [29]. We observed that spinetoram induced a 2.41-fold increase in *nAChRα6* expression relative to controls (2.41 ± 0.47-fold), while combined treatment further elevated this to 3.73 ± 0.22-fold (Figure 4(A)). These results indicate that upregulation of the target gene is associated with the enhanced insecticidal efficacy of the combined treatment. This suggests that elevated *nAChRα6* expression may render pests more susceptible to spinetoram, thereby contributing to the synergistic insecticidal effect. Analysis of metabolic resistance-associated enzyme activities showed that GST, CarE, POD, and CYP450 exhibited a time-dependent pattern over 24–72 h, characterized by an initial increase followed by a decline, with peak activity observed at 48 h (Figure 4(F,I)). Compared with the single treatment group, we observed that GST and POD activities in the combined treatment group were significantly higher during the 24–60 h but significantly lower at 72 h. CarE and CYP450 activities were significantly higher than in the single treatment group during the 24–48 h period but significantly lower during the 60–72 h period. These results showed that the combined treatment induced earlier and stronger responses of metabolic resistance-associated enzyme systems. Enzyme activities were significantly elevated at the early stage but declined rapidly after reaching peak levels. This dynamic pattern indicates that the short-term detoxification capacity of the western flower thrips has increased, but the subsequent rapid decline in enzyme activity suggests a decline in its detoxification capacity.

**Figure 4.**
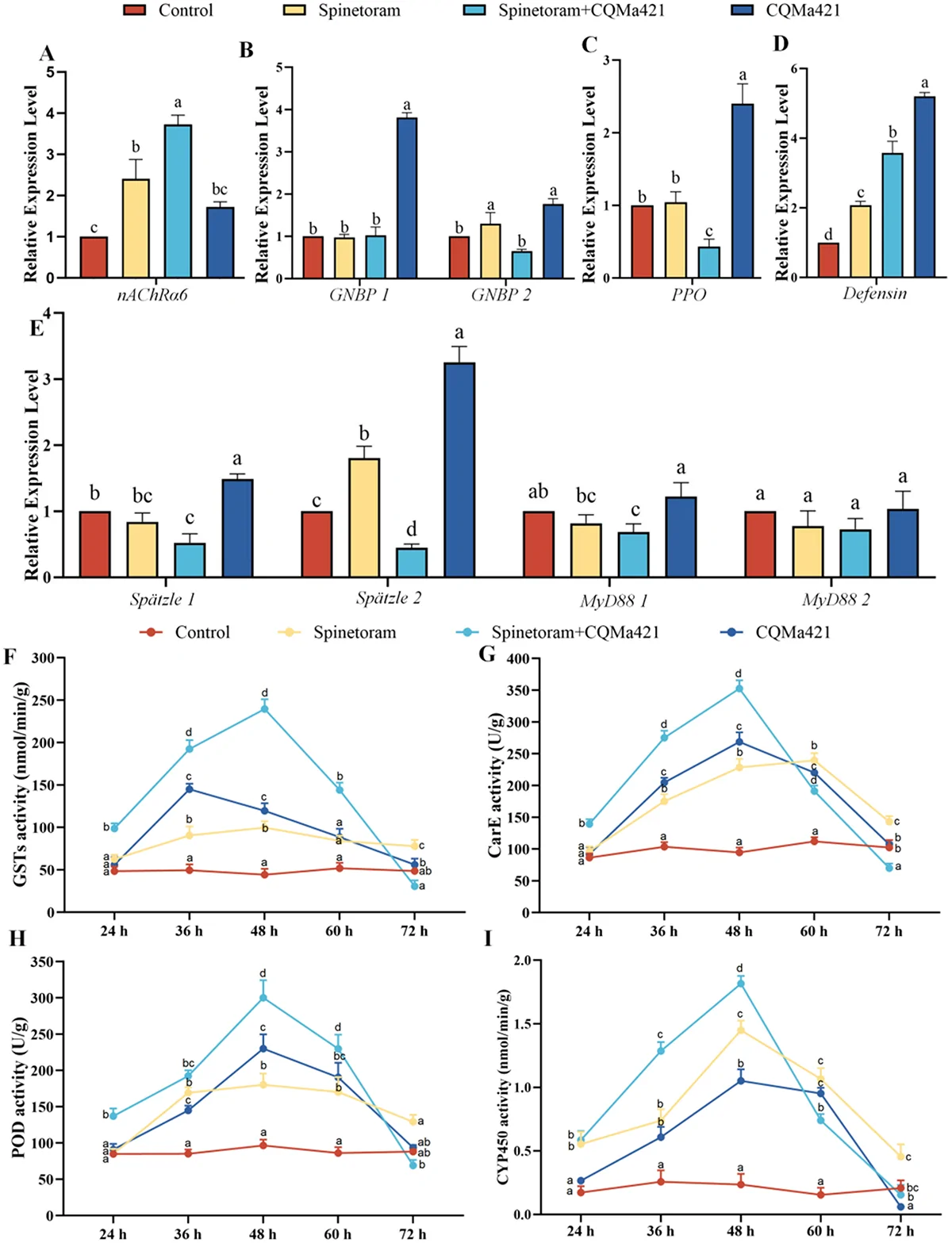
Effects of combined treatment with CQMa421 and spinetoram on expression of resistance target genes, immune responses, and detoxification responses in *F. occidentalis*.

Analysis of genes involved in immune recognition and signal transduction indicates that, compared with the CQMa421 group, significantly downregulated expression was observed in the combined treatment group for pattern recognition receptors *GNBP1/2*, key Toll pathway molecules *Spätzle1/2*, adaptor protein *MyD88 1*, the pro-phenoloxidase gene *PPO*, and the antimicrobial peptide gene *defensin* (Figure 4(B,E)). This indicates that combined treatment significantly suppressed the host’s immune response, including pathogen recognition, Toll-like receptor signaling, antimicrobial peptide synthesis, and the melanization response. Consequently, this may create favorable conditions for *M. anisopliae* to invade and colonize the host organism.

(A) *nAChRα6.* (B) *GNBP1/2*. (C) *PPO*. (D) *Defensin*. (E) *Spätzle1/2* and *MyD881/2* genes. (F) Glutathione S-transferase (GST) activity. (G) Carboxylesterase (CarE) activity. (H) Peroxidase (POD) activity. (I) Cytochrome P450 (CYP450) activity. Error bars denote standard deviation (SD) from the mean. Different lowercase letters indicate significant differences between groups (one-way ANOVA followed by Tukey’s HSD test, *p* < 0.05).

### *Spinetoram promotes germination and appressorium formation of CQMa421 on the cuticle of* F. occidentalis

We observed that the combined treatment of spinetoram and CQMa421 significantly accelerated the early infection process of CQMa421 on the body surface of the *F. occidentalis* (Figure 5(A)). Compared with the CQMa421 group, earlier spore germination and a significantly increased germination rate were observed in the combined treatment group (Figure 5(B)); the GT_50_ (time to 50% germination) was calculated to be 12.72 ± 0.33 h, representing a 24.01% reduction (Figure 5). The appressorium formation rate was also markedly elevated in the combined treatment group (Figure 5), with the AT_50_ (time to 50% appressorium formation) significantly reduced to 21.42 ± 0.87 h, a 17.90% decrease (Figure 5(E)). These findings demonstrate that the combined application of spinetoram and CQMa421 effectively promotes spore germination and appressorium formation of CQMa421 on the body surface of *F. occidentalis*, thereby accelerating its early infection events on the host cuticle.

**Figure 5.**
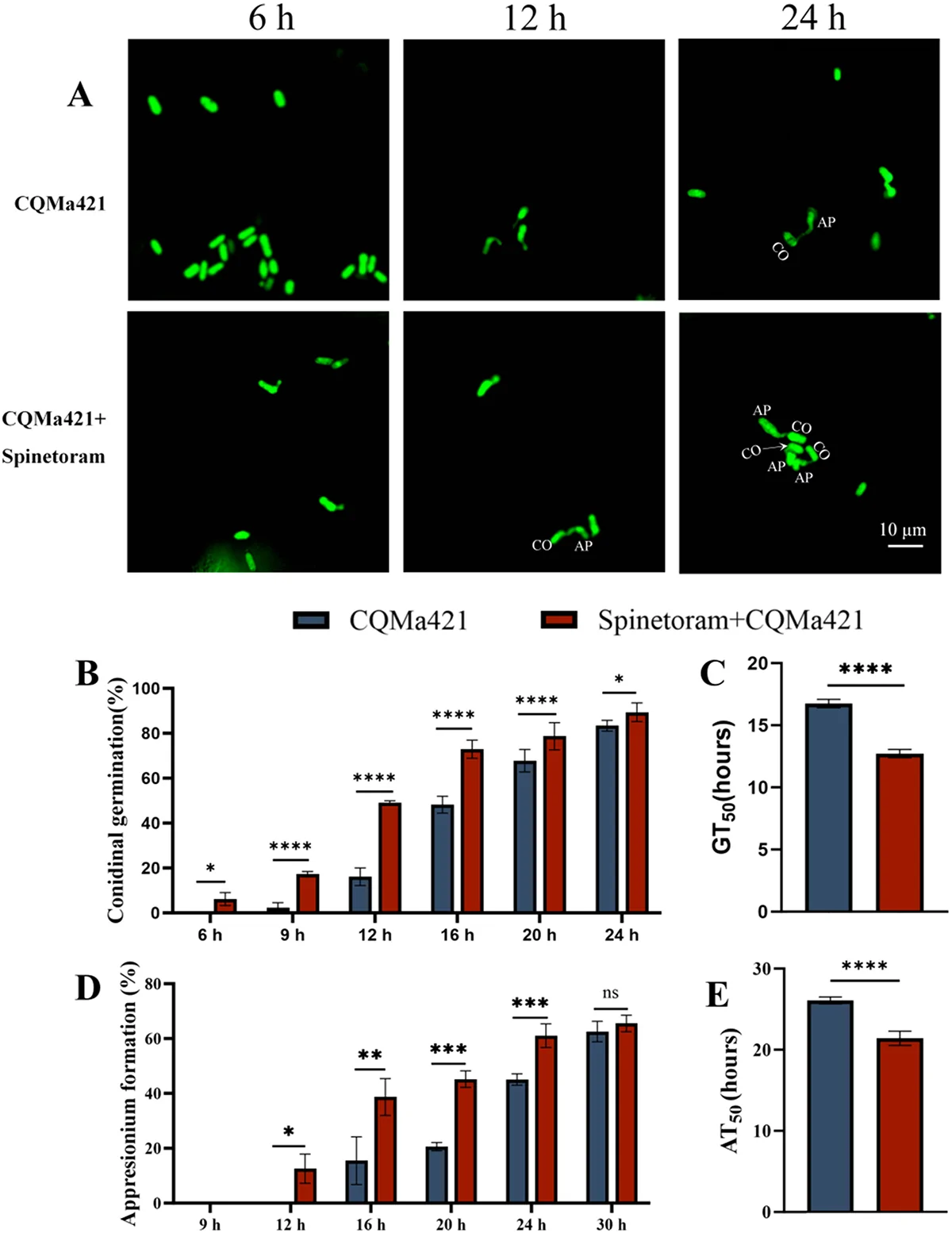
Spinetoram promotes the germination and appressorium formation of CQMa421 on the cuticle of *F. occidentalis*.

(A) Fluorescent micrographs illustrating germination conidia (CO) and appressorium formation(AP), Scale bars: 10 μm. (B) Germination rate of CQMa421. (C) Germination time (GT_50_) of CQMa421. (D) Appressorium formation rate of CQMa421. (E) Appressorium formation time (AT_50_) of CQMa421. *t-test*: *p < 0.05, **p < 0.01, ***p < 0.001; ns denotes no statistically significant difference.

### *Field efficacy trial of CQMa421 combined with spinetoram against* F. occidentalis

Our results showed that the combined treatment group achieved a control efficacy of 90.67% ± 3.05% on the third day post-application. This was significantly higher than that of CQMa421 (52.67% ± 2.51%), the 10.0 mg/L spinetoram (57.33% ± 4.75%), and the 20.0 mg/L spinetoram (74.33% ± 4.04%) (one-way ANOVA, Tukey’s test, *p* < 0.001, *n* = 3). At 14 d post-application, a control efficacy of 86.33% ± 4.75% was maintained in the combined treatment group, which was significantly higher than the 43.33% ± 4.72% observed in the 20.0 mg/L spinetoram and the 65.67% ± 2.88% recorded for the CQMa421 group (Table 5). These findings demonstrate that, under the conditions of this field trial, the combination of CQMa421 and spinetoram offers both rapid and persistent efficacy, providing a reliable strategy to reduce pesticide use while enhancing effectiveness.

**Table 5.** Field control efficacy of CQMa421 combined with spinetoram against *F. occidentalis* in field conditions.

Insecticides applied	Concentration of CQMa421 (conidia/mL)	Concentration of spinetoram (mg/L)	Time		
			1 days	3 days	14 days
CQMa421 + spinetoram	10^7^	10	74.67 ± 4.16a	90.67 ± 3.05a	86.33 ± 4.75a
spinetoram	0	10	51.67 ± 5.86b	57.33 ± 4.75c	36.67 ± 1.52c
spinetoram	0	20	71.33 ± 3.05a	74.33 ± 4.04b	43.33 ± 4.72c
CQMa421	10^7^	0	28.67 ± 2.51c	52.67 ± 2.51c	65.67 ± 2.88b

### *The effect of combined treatment on the control of* F. occidentalis *throughout the growth period of aubergines*

From April to September 2024, field trials were conducted throughout the entire growth period of eggplant in Bishan District, Chongqing, to evaluate the efficacy of CQMa421 and spinetoram against *F. occidentalis*. In response to the dynamic occurrence of *F. occidentalis* during the experimental period, 7 applications of the spinetoram (20 mg/L) and four applications of the combined treatment (1 × 10^7^ conidia/mL CQMa421 + 10 mg/L spinetoram) were administered during the peak infestation months of June to August (Figure 6). The results showed that, during the peak occurrence period from June to August, the combined treatment group consistently maintained lower population densities (2.3–6.3 individuals per flower). In contrast, the population density in the 20 mg/L spinetoram group ranged from 6.4 to 14 individuals per flower, which was significantly higher than that of the combined treatment group during the same period. These findings indicate that the combined prevention and control strategy holds great potential for widespread application.

**Figure 6.**
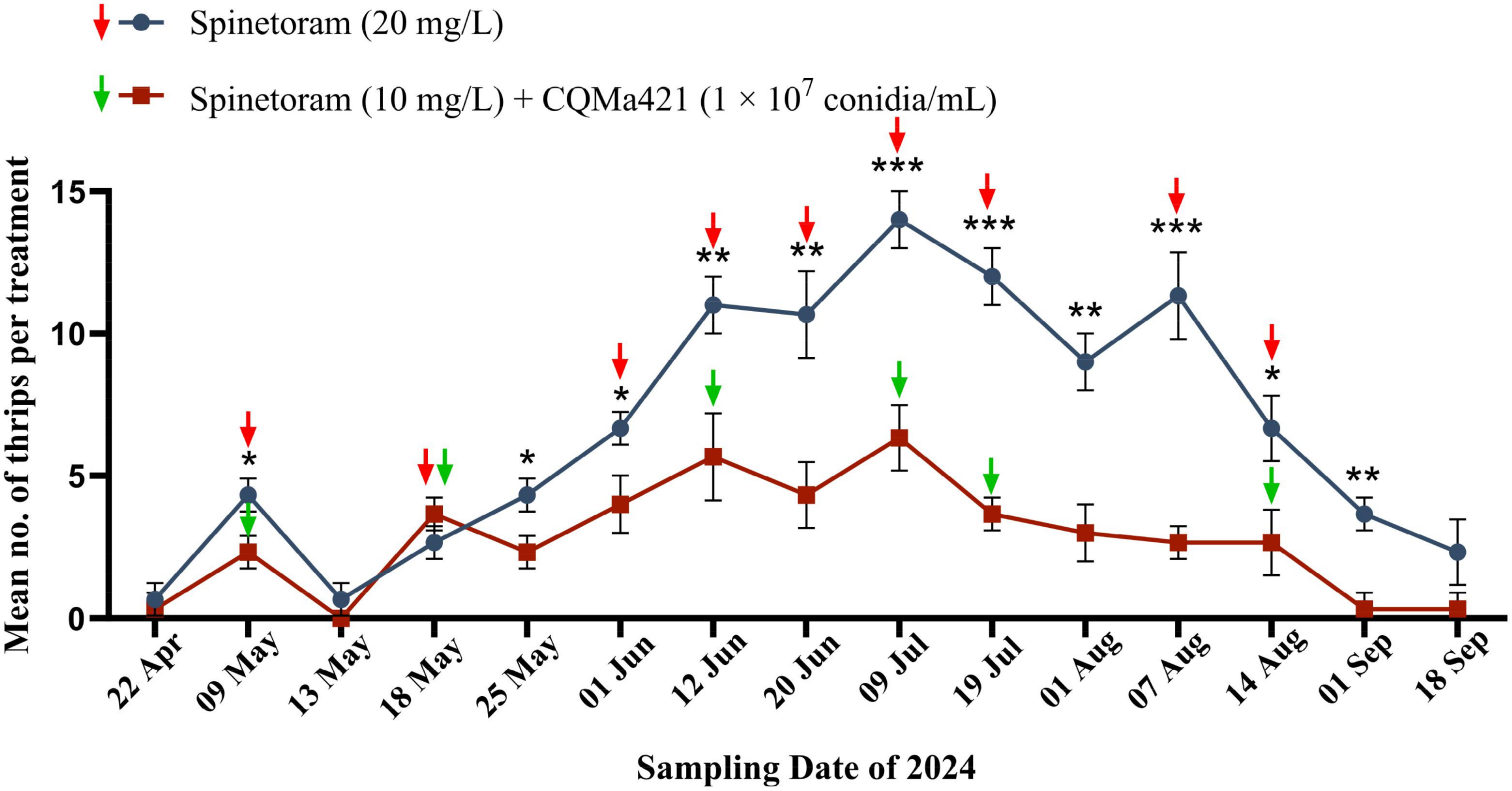
Number of *F. occidentalis* per flower in the 2024 demonstration field trial. In the 2024 field trials, the mean number of *F. occidentalis* per flower (mean ± SD). *t-test*: *p < 0.05, **p < 0.01, ***p < 0.001. The position of the red or green arrow indicates the timing of pesticide application at that point.

## Discussion

*F. occidentalis* threatens global agriculture, causing economic losses and transmitting plant viruses [30]. Overuse of broad-spectrum insecticides, especially spinetoram, has increased resistance in *F. occidentalis* and reduced control efficacy [31]. Combining entomopathogenic fungi with chemical insecticides enhances pest control, offering a sustainable management approach [32–34]. *M. anisopliae* CQMa421 (registration No. PD20171744), a broad-spectrum entomopathogenic fungus, is registered in China against various pests, including *F. occidentalis*, rice planthoppers, and rice leaf rollers. Field applications have demonstrated its excellent sustained control efficacy. However, in practical applications, CQMa421 is highly dependent on environmental conditions, a factor that limits its widespread adoption [35,36]. In this study, the combination of CQMa421 with a 50% reduced dose of spinetoram was found to exhibit a synergistic effect on *F. occidentalis* mortality, while simultaneously suppressing second-generation thrips populations. This combination showed rapid and sustained insecticidal activity, indicating its significant application potential within integrated pest management (IPM) strategies. These findings substantiate the advantages of combining entomopathogenic fungi with chemical insecticides for pest control. However, in this experiment, 5% emamectin benzoate from different manufacturers differentially affected *M. anisopliae* CQMa421 spore germination. This discrepancy likely stems from factors beyond the active ingredient, including adjuvant types and concentrations and manufacturing process variations, all significantly affecting fungal spore germination. Therefore, in practice, compatibility between specific insecticides and entomopathogenic fungi must be tested.

Some chemical insecticides synergize with entomopathogenic fungi via physiological disruption, immune suppression, or behavioral alteration [37]. For example, imidacloprid enhances *B. bassiana* infection in *Reticulitermes flavipes* by interfering with feeding, grooming, and immune behaviors [38]; organophosphorus insecticides promote fungal infection via immune suppression in *Leptinotarsa decemlineata* [39]; and emamectin benzoate increases *M. anisopliae* CQMa421 pathogenicity by weakening immune barriers and delaying molting in *S. frugiperda* [22]. Entomopathogenic fungi infect hosts through cuticle penetration, in vivo proliferation, and toxin secretion [40], disrupting cuticle integrity, metabolism, neural function, and immunity to increase insecticide susceptibility [41,42]. In this study, *M. anisopliae* CQMa421 and spinetoram exhibited synergistic toxicity against *F. occidentalis* via reciprocal enhancement. Co-application with CQMa421 reduced the spinetoram LC_50_ by 3.80- and 4.54-fold relative to spinetoram alone, indicating significantly enhanced spinetoram susceptibility in *F. occidentalis*. Spinetoram is a nicotinic acetylcholine receptor (nAChR) agonist with *nAChRα6* as its core functional target, inducing persistent neural excitation and paralysis [43]. Our results showed that the combined treatment significantly upregulated the transcript abundance of *nAChRα6*. Previous functional studies have confirmed that loss-of-function mutations or knockout of *nAChRα6* confer high-level resistance to spinosyns, including spinetoram [44,45]. It is thus hypothesized that the upregulated expression of *nAChRα6* under the combined treatment may theoretically expand the pool of functional spinosyn-sensitive targets in insects, thereby potentiating the neurotoxic efficacy of spinetoram. Notably, *nAChRα6* modulation is not the sole mediator of this synergistic effect. The combined treatment also disrupted activities of key detoxification enzymes (GST, CarE, POD, CYP450), suggesting impaired metabolic detoxification capacity. Furthermore, the entire host immune cascade-from immune recognition (*GNBP*) and Toll pathway signal transduction to effector molecules-was comprehensively suppressed, which may further facilitate fungal colonization and improve field control efficacy.

In the present study, GST, CarE, and POD all showed an initial increase followed by a decline under both single and combined treatments. This pattern is consistent with the enzymatic responses reported in *Spodoptera litura* infected with *Metarhizium rileyi* [46] and in white-backed planthoppers exposed to sublethal thiamethoxam [47], suggesting that compensatory upregulation of detoxification enzymes is a common metabolic response to single stress. Among these enzymes, POD showed a broadly similar rise-and-fall pattern across different fungus-pesticide combinations [32,39], indicating that oxidative stress responses may be relatively conserved. By contrast, GST and CarE responded more variably. For example, in locusts treated with *M. anisopliae* and chlorantraniliprole, both enzymes were initially inhibited [32], whereas they first increased and then declined in this study. Such differences may reflect the substrate specificity of detoxification enzymes and their sensitivity to pathogen species, pesticide mode of action, and host physiological state.

Current assessments of fungal-insecticide synergies focus on immediate pest populations, ignoring effects on the offspring of survivors. This results in incomplete assessments of synergistic efficacy. Research shows that *Isaria javanica* infection significantly reduces pupation rate and total egg production in surviving *Sogatella furcifera* [48], indicating that entomopathogenic fungi substantially diminish host reproductive capacity [49,50]. Spinetoram has also been demonstrated to reduce the egg-laying rates of numerous insect species [51,52]. This study examined combined spinetoram and *M. anisopliae* effects on the current-generation *F. occidentalis* and, via systematic analysis, on survivor reproduction and second-generation population dynamics. The combined treatment synergistically suppressed second-instar nymph, pupa, and adult numbers and emergence rates, and reduced pupation rates. Adult thrips cumulative numbers decreased by over 50% compared to single treatment, demonstrating sustained population suppression. Field trials validated this synergy: the combined treatment outperformed spinetoram at its recommended dose, with 50% reduced spinetoram and 33% fewer applications. The field trial was conducted at a single site over one growing season in Chongqing. The observed control efficacy should be interpreted within the trial’s specific environmental and agronomic conditions and is not representative of all production regions or seasons. Both fungal control efficacy and spinetoram persistence are affected by environmental factors and application practices, so the synergistic effects of their combination may vary by region, season, and cropping system. Multi-site, multi-season field trials are therefore required to validate the robustness and broader applicability of this strategy. Nevertheless, consistent synergistic effects across laboratory and field trials provide preliminary evidence supporting this control strategy.

## Conclusions

This study shows that *M. anisopliae* CQMa421 combined with a 50%-reduced spinetoram acts synergistically against *F. occidentalis*, enhances the pest’s susceptibility to spinetoram, and suppresses its second-generation population. The combined treatment upregulates the resistance target *nAChRα6*, while disrupting metabolic resistance-associated enzymes (GST, CarE, POD, and CYP450), indicating dysregulation of *F. occidentalis* metabolic defense. Furthermore, it suppresses immune genes (*GNBP1/2*, *Spätzle1/2*, *MyD881*, *Defensin*, *PPO*), thereby weakening host defense and promoting early fungal infection. In field trials, the combined treatment remained more effective and persistent than chemical pesticides despite reduced application frequency and dosage. These findings elucidate the synergistic mechanisms underlying reciprocal enhancement, offering a basis for green pest control and reduced chemical use.

## Supplementary Material

Table S1.xlsx

## Data Availability

The datasets generated and analyzed during the current study are openly available on Figshare at https://doi.org/10.6084/m9.figshare.32306793 [53].
